# Author Correction: Influence of El Niño on the variability of global shoreline position

**DOI:** 10.1038/s41467-023-40239-4

**Published:** 2023-07-25

**Authors:** Rafael Almar, Julien Boucharel, Marcan Graffin, Gregoire Ondoa Abessolo, Gregoire Thoumyre, Fabrice Papa, Roshanka Ranasinghe, Jennifer Montano, Erwin W. J. Bergsma, Mohamed Wassim Baba, Fei-Fei Jin

**Affiliations:** 1 LEGOS (Université de Toulouse/CNRS/IRD/UPS), Toulouse, France; 2 Department of atmospheric sciences (University of Hawaii at Manoa), Honolulu, USA; 3 Ecosystems and Fishery Resources Laboratory, Institute of Fisheries and Aquatic Sciences, University of Douala, Douala, Cameroon; 4 Universidade de Brasília (UnB), IRD, Instituto de Geociencias, Brasilia, Brazil; 5 Department of Coastal and Urban Risk & Resilience, IHE Delft Institute for Water Education, P.O. Box 3015, 2610 DA Delft, The Netherlands; 6 Harbour. Coastal and Offshore Engineering, Deltares, PO Box 177, 2600 MH Delft, The Netherlands; 7 Water Engineering and Management, Faculty of Engineering Technology, University of Twente, PO Box 217, 7500 AE Enschede, The Netherlands; 8 GET (Université de Toulouse/CNRS/IRD/UPS), Toulouse, France; 9 Earth Observation Lab, French Space Agency (CNES), Toulouse, France; 10 Center for Remote Sensing Application (CRSA), Mohammed VI Polytechnic University (UM6P), Ben Guerir, 43150 Morocco

**Keywords:** Natural hazards, Physical oceanography

Correction to: *Nature Communications* 10.1038/s41467-023-38742-9, published online 12 June 2023

The original version of this Article contained an error in Fig. 3, in which figure panels (i)–(n) were replicas of panels (c)–(h). Panels (i)–(n) have now been replaced by the correct figure panels. This has been corrected in both the PDF and HTML versions of the Article.

