# Supplementary material for: Influence of El Niño on the variability of global shoreline position
Source: Nat Commun. 2023 Jun 12;14:3133. doi: 10.1038/s41467-023-38742-9 (PMC10261116; doi:10.1038/s41467-023-38742-9)
Supplement: Supplementary file 1 — Supplementary Information [file 41467_2023_38742_MOESM1_ESM.pdf]

SUPPLEMENTARY MATERIAL for

Influence of El Niño on the variability of global shoreline position

Rafael Almar[1,*], Julien Boucharel[1,2,*], Marcan Graffin[1], Gregoire Ondoa Abessolo[3], Gregoire Thoumyre[1], Fabrice Papa[1,4], Roshanka Ranasinghe[5,6,7], Jennifer Montano[8], Erwin W.J. Bergsma[9], Mohamed Wassim Baba[10] and Fei-Fei Jin[2]

[1]LEGOS (Université de Toulouse/CNRS/IRD/UPS), Toulouse, France

[2]Department of atmospheric sciences (University of Hawaii at Manoa), Honolulu, USA

[3]Ecosystems and Fishery Resources Laboratory, Institute of Fisheries and Aquatic Sciences, University of Douala, Cameroon

[4]Universidade de Brasília (UnB), IRD, Instituto de Geociencias, Brasilia, Brazil

[5]Department of Coastal and Urban Risk & Resilience, IHE Delft Institute for Water Education, P.O. Box 3015 2610 DA Delft, The Netherlands

[6]Harbour. Coastal and Offshore Engineering, Deltares, PO Box 177, 2600 MH Delft, The Netherlands

[7]Water Engineering and Management, Faculty of Engineering Technology, University of Twente, PO Box 217, 7500 AE Enschede, The Netherlands

[8]GET (Université de Toulouse/CNRS/IRD/UPS), Toulouse, France

[9]Earth Observation Lab, French Space Agency (CNES), Toulouse, France

[10]Center for Remote Sensing Application (CRSA), Mohammed VI Polytechnic University (UM6P), Ben Guerir, 43150, Morocco

[*]e-mail: rafael.almar@ird.fr; bouch@hawaii.edu

Figure S1 provides a schematic conceptualization of the approach undertaken in the manuscript.

Figure S2 shows the seasonally modulated ENSO teleconnections patterns for the two types of El Niño events.

Figure S3 shows correlation scores between observed and simulated shoreline drivers using the complex ENSO based model. Results are also presented latitudinally and longitudinally averaged to demonstrate the complex ENSO's influence on remote oceanic basins and at high latitudes as compared to its canonical expression and to other modes of extratropical climate variability (NAO and SAM).

We can observe ENSO influence not only in the tropical Pacific, its dominant region of influence previously as shown[1], but also at higher latitudes and in other oceanic basins. Figure S3 exhibits latitudinal disparities among the different drivers of shoreline variability (Fig. S3b,e,h) that can be attributed in part to the geographical land mass distribution. In particular, although air-sea interactions certainly play a role, latitudinal asymmetries of the land-sea distribution and related radiative hemispheric imbalance are known to be the main reason for the perennial position of the Inter Tropical Convergence Zone (ITCZ) in the Northern Hemisphere[2]. This large-scale atmospheric feature characterized by heavy precipitations is strongly impacted by ENSO variability, which explains the overall increased coherence between ENSO and river discharge evolution between 15ºN and 30ºN where the ITCZ is located (Fig. S3h). In contrast, we observe a larger correlation between SLA and ENSO variability in the Southern Hemisphere (30ºS-50ºS) than in the Northern Hemisphere, likely due to the ENSO influence that extends along the South American shorelines (Fig. S3b). The ENSO control on coastal wave activity is rather evenly distributed latitudinally with a single peak in the tropical belt as a result of the seasonal ENSO influence on both tropical and extratropical storm activity. Yet, a small secondary peak with increased correlations between 5ºN and 30ºN (Fig. S3e) can be observed, which can be explained by the presence of the three most active Tropical Cyclone basins in the Northern Hemisphere[3,4,5].

The classic Niño3 (SST anomalies averaged in the region 5ºN-5ºS, 170ºW-120ºW) and E-index and also Niño4 (SST anomalies averaged in the region 5ºN-5ºS, 160ºE-150ºW) and C-index are respectively nearly identical and they offer a simpler and more direct measure of ENSO spatial diversity, which indeed does not require an EOF analysis[6]. Therefore, as a consistency check, we re-ran both Figure 2 and Figure 3 using the classical Niño indices, which are presented in Figure S4 and S5 respectively. The results are unchanged, except that the variance explained by Niño3 is greater than that explained by the E index for all coastal drivers, which is likely due to the fact that the E index tends to capture mostly extreme events (such as the 1997/98 and 2015/16 ENSO events), whereas Niño3 captures more canonical ENSO variability.

Figure S6 shows comparison of shorelines detected from satellite imagery using our auto-detection algorithm and in-situ measurements of shorelines at 4 locations around the world.

Figure S7 shows the decorrelation time-scales of shoreline and its drivers at interannual time scales used to infer threshold for statistically significant correlation at the 95% confidence level based on a Student *t*-test.

Figure S8 shows the local lead-lag analysis (months of the maximum correlation between shoreline and its dominant drivers interannual variability).

Table S1 shows global and tropical averaged correlation coefficients and the proportion of shorelines where correlations are statistically significant at the 95% level between observed and simulated variables (SLA, Wave energy flux, River flow and Shoreline) with different climate-based multi-regression models (canonical ENSO, complex ENSO and all climate modes).

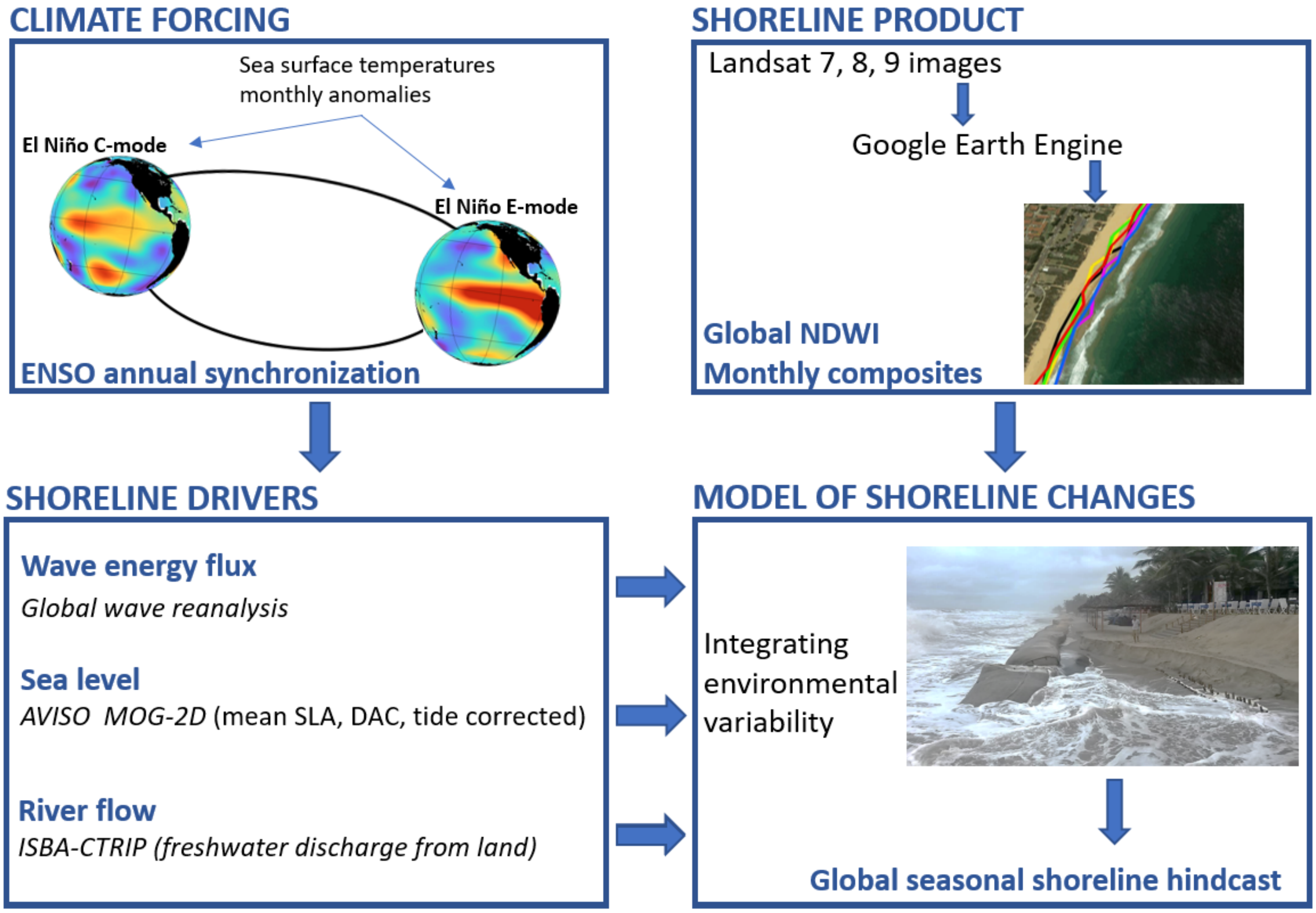

Figure S1. *Schematic diagram of the methodological approach adopted in this study. The ENSO maps in upper left panel were generated by J. Boucharel, the satellite image in upper right panel was extracted from Google Earth (2023) with data and background images from TerraMetrics, SIO, NOAA, US Navy, NGA and GEBCO (Narrabeen, Australia), and the lower right picture was taken by R. Almar at Hoi An, Vietnam (2017).*

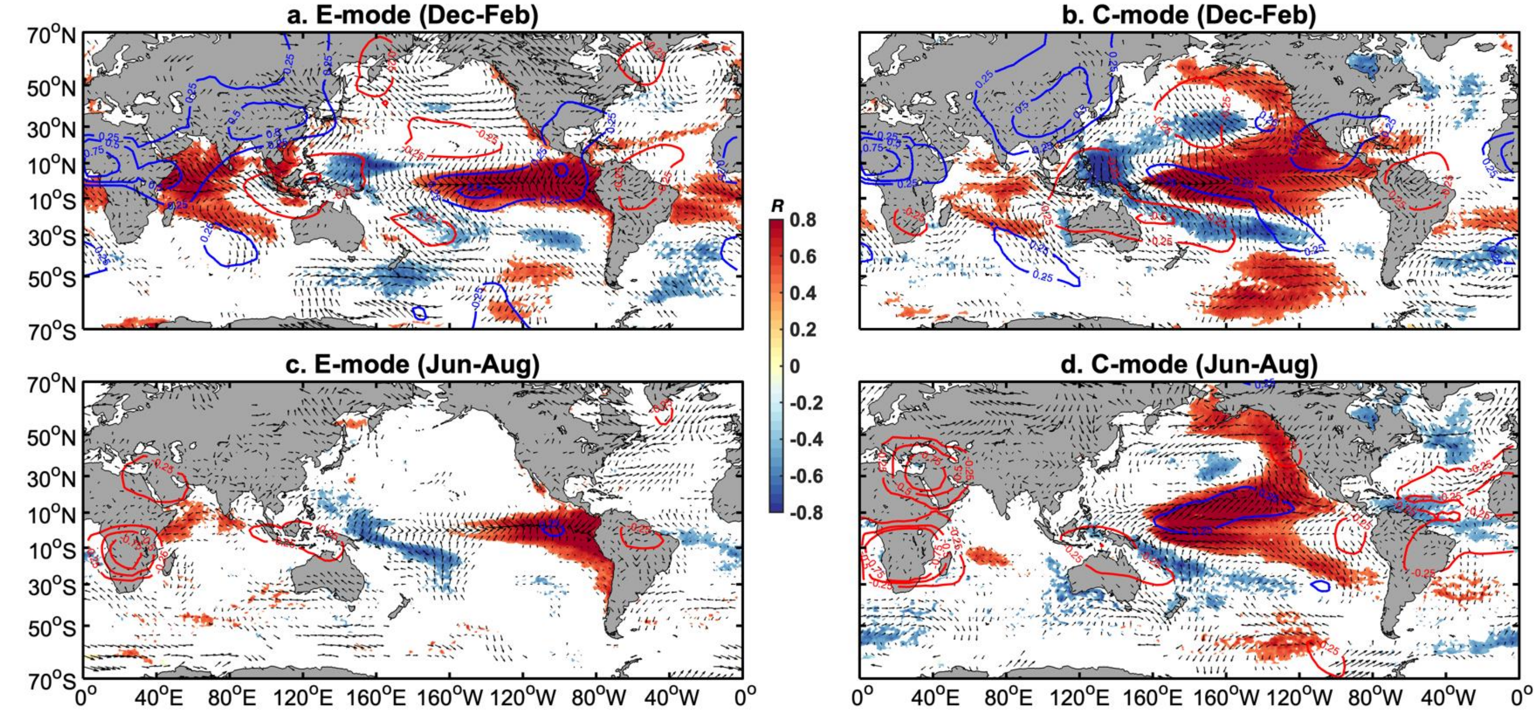

Figure S2. ***Diversity of ENSO's seasonal teleconnections.*** *Regression patterns between interannual anomalies of precipitation (blue/red contours for wet/dry), sea surface temperature (shading), wind speed and direction (arrows) and the E-mode (left panels) and C-mode (right panels) during boreal winters (top panels) and summers (bottom panels).*

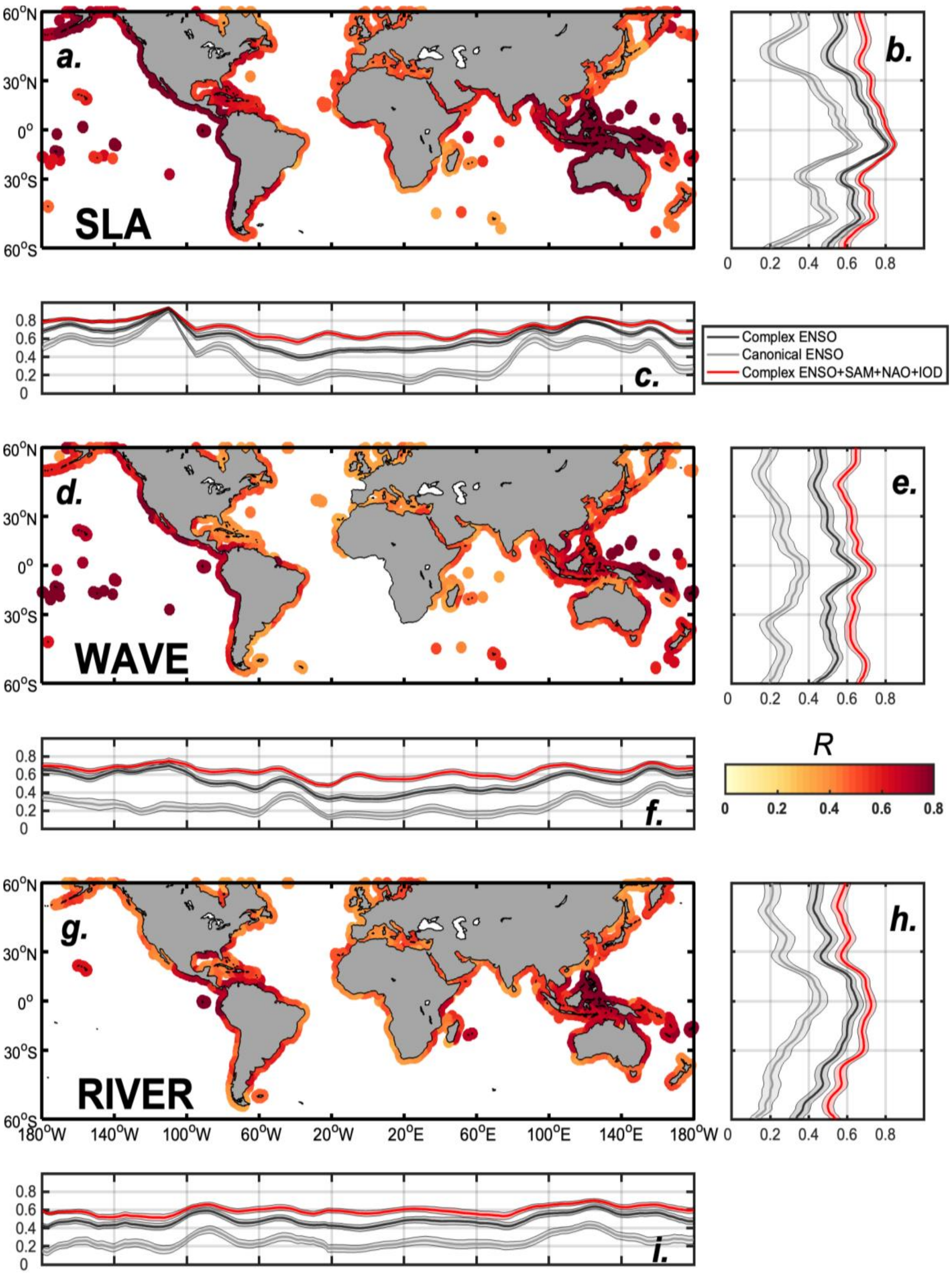

Figure S3. ***Complex ENSO influence on drivers of shoreline change.*** *(a.) Global distribution of correlation coefficients between observed and simulated ENSO-based yearly anomalies of sea level. (b.) Latitudinal distribution of longitudinally averaged correlations between observed SLA and ENSO-based model (black), a climate-based model that also includes effects from SAM and NAO (red) and a simple linear regression model onto Niño3 (representing only the canonical ENSO mode in grey). (c., Longitudinal distribution of meridionally averaged correlations between observed SLA and ENSO-based (black), climate-based (red) and the canonical ENSO model (grey) for sea level. (d.), (e.) and (f.) are respectively the same as (a.), (b.) and (c.) for wave energy. (g.), (h.) and (i.) are respectively the same as (a.), (b.) and (c.) for river flow. Shading delineates the range of one standard deviation among all randomized hindcasts of varying lengths from 10 to 17 years. The small black crosses in panels (a.), (d.) and (g.) indicate portions of shoreline where correlations are below the 95% confidence threshold.*

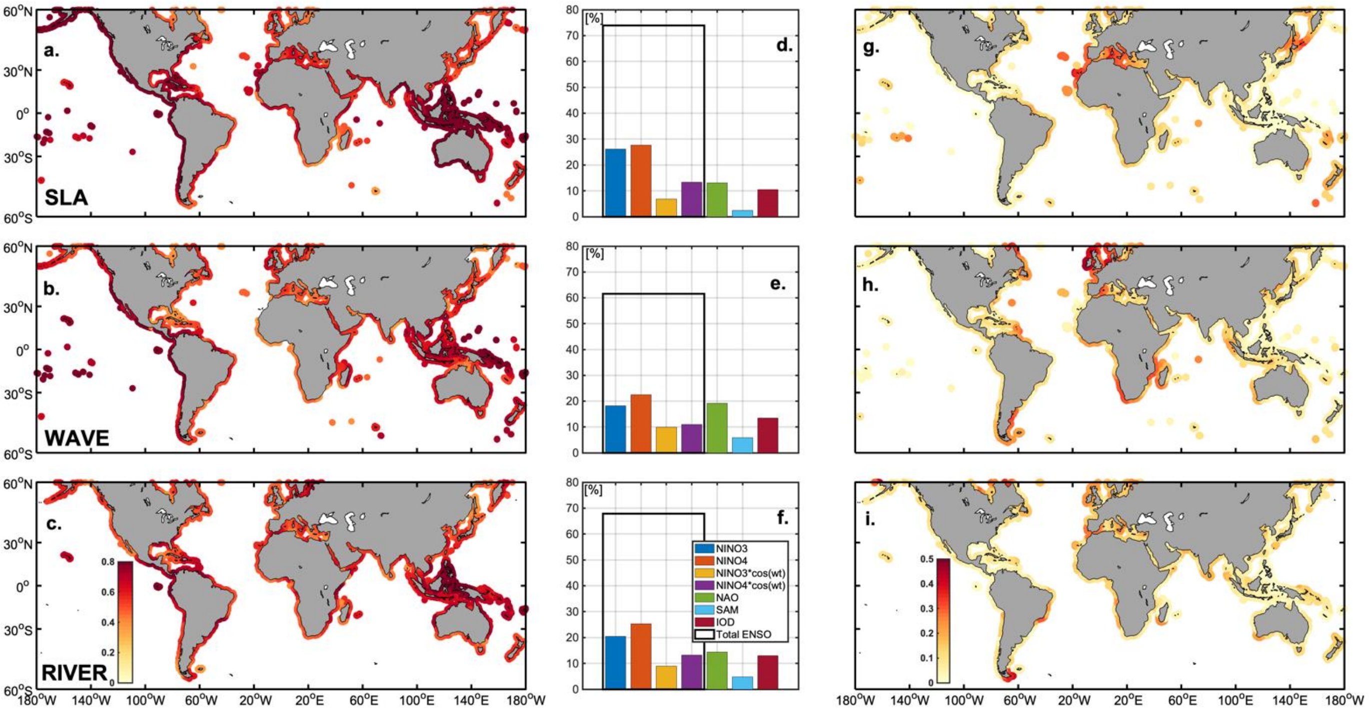

Figure S4. *Same as Figure 2 from the manuscript but using Niño3 and Niño4 indices instead of the E and C indices respectively.*

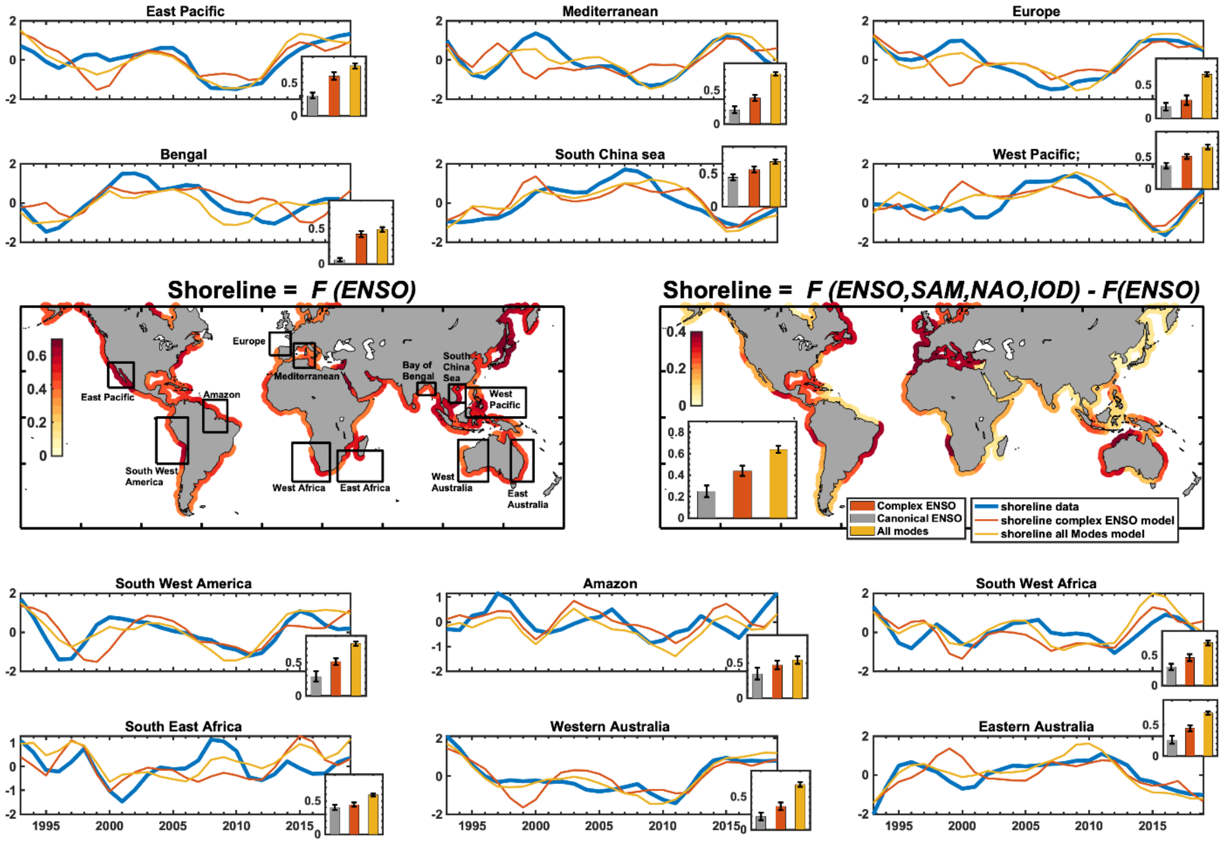

138 **Figure S5.** *Same as Figure 3 from the manuscript but using Niño3 and Niño4 indices instead of the E and C indices respectively.*

139

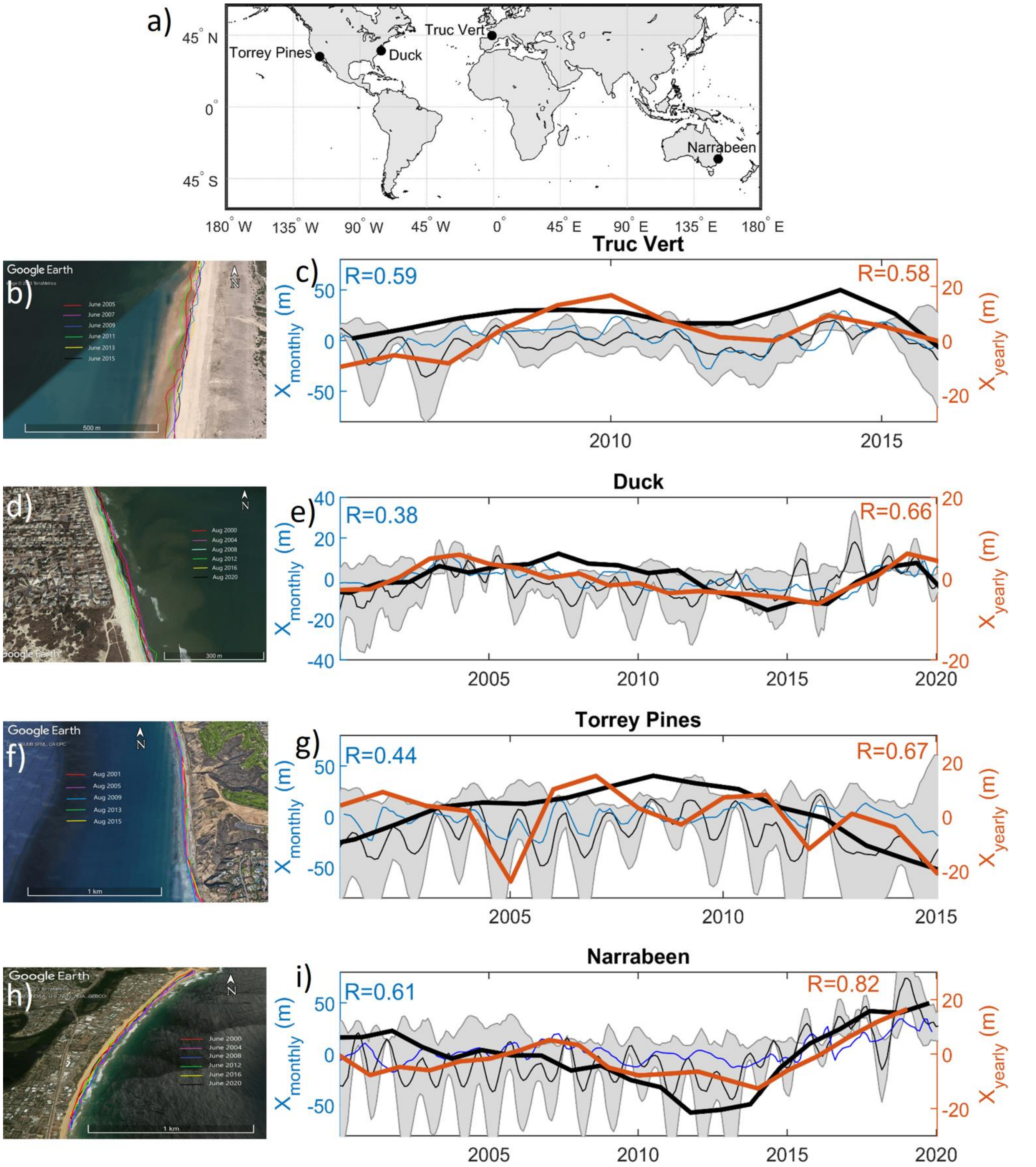

Figure S6. *Comparison of shorelines detected from satellite imagery using our auto-detection algorithm and in-situ measurements of shorelines at 4 locations around the world. a): Locations of the different sites from where in-situ measurements were used for the analysis; b) and c) Truc Vert[7], France; d) and e) Duck, USA(data provided by the U.S. Army Engineer Research & Development Centre, Coast & Hydraulics Laboratory, Field Research Facility); f) and g) Torrey Pines[8], USA and h) and i) Narrabeen[9], Australia. First column shows illustrations of Google earth images on which satellite derived shorelines at selected times are superimposed. The second column shows the in-situ data (colored lines) and the shorelines extracted from satellite images (black lines) at the closest computational coastal point. Thin lines show the monthly time series and thick lines the yearly time series. Grey shading indicates the geographical standard deviation within 400 km around the closest satellite-derived transect. Panels b), d), f, h) are extracted from Google Earth (2023) with data and background images from TerraMetrics, CSUMB SFML, CA OPC, and SIO, NOAA, US Navy, NGA and GEBCO.*

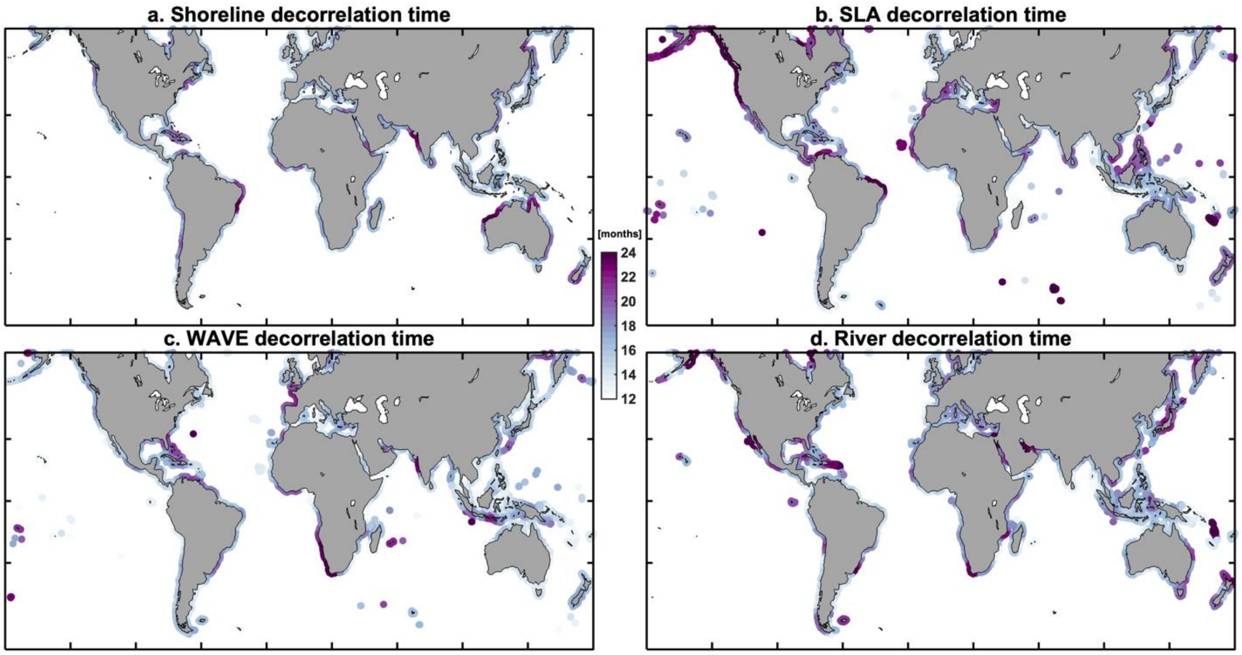

Figure S7. *Decorrelation time of Shoreline (a.), SLA (b.), Wave energy (c.) and River discharge (d.) interannual anomalies.*

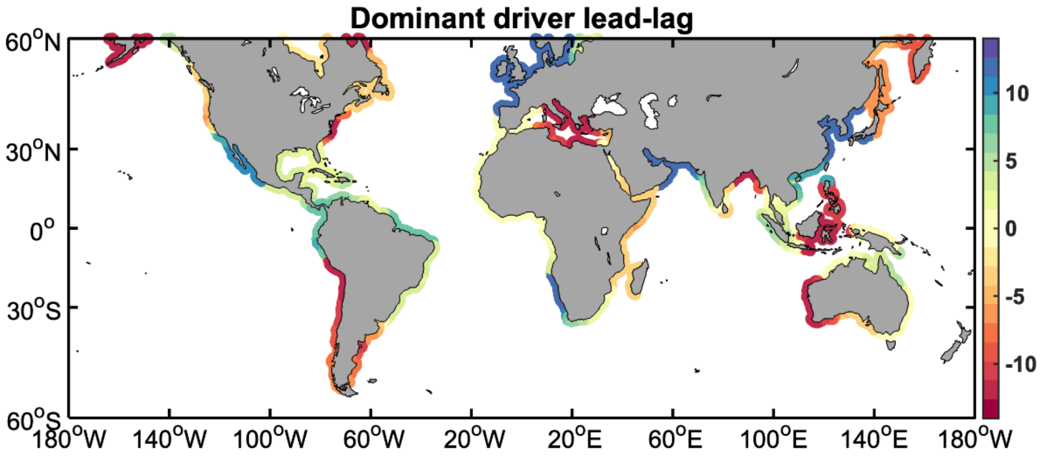

Figure S8. *Local lead-lag analysis (months of the maximum correlation between shoreline and its dominant drivers interannual variability).*

	All Modes				Complex ENSO				Canonical ENSO			
	Global		Tropics		Global		Tropics		Global		Tropics	
**SLA**	0.72	100%	0.78	100%	0.63	99%	0.72	99%	0.42	52%	0.54	72%
**WAVE**	0.64	100%	0.66	100%	0.50	94%	0.56	99%	0.25*	22%	0.32	41%
**RIVER**	0.61	100%	0.67	100%	0.51	98%	0.59	99%	0.29*	37%	0.38	64%
**Shoreline**	0.64	100%	0.63	100%	0.47	84%	0.47	93%	0.25*	17%	0.29*	31%

Table S1. *Global (black font) and tropical (23ºS-23ºN in red font) average of correlation coefficients between observed and simulated interannual anomalies of sea level, wave energy flux, river flows and shoreline evolution as well as percentage of shorelines where the correlation is significant above the 95% confidence level when the multi-linear model considers all climate modes, the complex ENSO or the canonical ENSO only. The asterisks indicate correlations below the 95% statistical level of confidence.*

References

1. Vos, K., Harley, M. D., Turner, I. L., & Splinter, K. D. (2023). Pacific shoreline erosion and accretion patterns controlled by El Niño/Southern Oscillation. *Nature Geoscience*, *16*(2), 140-146.
2. Philander, S. G. H., Gu, D., Lambert, G., Li, T., Halpern, D., Lau, N. C., & Pacanowski, R. C. (1996). Why the ITCZ is mostly north of the equator. Journal of climate, 9(12), 2958-2972.
3. Lin, I. I., Camargo, S. J., Patricola, C. M., Boucharel, J., Chand, S., Klotzbach, P., ... & Jin, F. F. (2020). ENSO and tropical cyclones. *El Niño Southern Oscillation in a Changing Climate*, 377-408
4. Knutson, T., Landsea, C., & Emanuel, K. (2010). Tropical cyclones and climate change: A review. Global perspectives on tropical cyclones: from science to mitigation, 243-284.
5. Seneviratne, S. I., X. Zhang, M. Adnan, W. Badi, C. Dereczynski, A. Di Luca, S. Ghosh, I. Iskandar, J. Kossin, S. Lewis, F. Otto, I. Pinto, M. Satoh, S. M. Vicente-Serrano, M. Wehner, B. Zhou, 2021, Weather and Climate Extreme Events in a Changing Climate. In: Climate Change 2021: The Physical Science Basis. Contribution of Working Group I to the Sixth Assessment Report of the Intergovernmental Panel on Climate Change [Masson-Delmotte, V., P. Zhai, A. Pirani, S. L. Connors, C. Péan, S. Berger, N. Caud, Y. Chen, L. Goldfarb, M. I. Gomis, M. Huang, K. Leitzell, E. Lonnoy, J. B. R. Matthews, T. K. Maycock, T. Waterfield, O. Yelekçi, R. Yu and B. Zhou (eds.)]. Cambridge University Press. In Press.
6. Ren, H.L, and F.F. Jin (2011), Niño indices for two types of ENSO, Geophys. Res. Lett., 38.
7. Castelle, B., Bujan, S., Marieu, V. & Ferreira, S. 16 years of topographic surveys of rip-channelled high-energy mesomacrotidal sandy beach. Sci. Data DOI: 10.1038/s41597-020-00750-5 (2020).
8. Ludka, B., Guza, R., O'Reilly, C. & Merrifield, M. Sixteen years of bathymetry and waves at san diego beaches. Sci. Data DOI: 10.1038/s41597-019-0167-6 (2019).

9. Turner, I. L. et al. A multi-decade dataset of monthly beach profile surveys and inshore wave forcing at Narrabeen, Australia. Sci. Data 3 160024 DOI: 10.1038/sdata.2016.24 (2016).
